# Hemodynamic and Tubular Changes Induced by Contrast Media

**DOI:** 10.1155/2014/578974

**Published:** 2014-02-11

**Authors:** Antonella Caiazza, Luigi Russo, Massimo Sabbatini, Domenico Russo

**Affiliations:** Department of Surgery and Nephrology, University of Naples “Federico II”, Via Sergio Pansini 5, 80131 Naples, Italy

## Abstract

The incidence of acute kidney injury induced by contrast media (CI-AKI) is the third cause of AKI in hospitalized patients. Contrast media cause relevant alterations both in renal hemodynamics and in renal tubular cell function that lead to CI-AKI. The vasoconstriction of intrarenal vasculature is the main hemodynamic change induced by contrast media; the vasoconstriction is accompanied by a cascade of events leading to ischemia and reduction of glomerular filtration rate. Cytotoxicity of contrast media causes apoptosis of tubular cells with consequent formation of casts and worsening of ischemia. There is an interplay between the negative effects of contrast media on renal hemodynamics and on tubular cell function that leads to activation of renin-angiotensin system and increased production of reactive oxygen species (ROS) within the kidney. Production of ROS intensifies cellular hypoxia through endothelial dysfunction and alteration of mechanisms regulating tubular cells transport. The physiochemical characteristics of contrast media play a critical role in the incidence of CI-AKI. Guidelines suggest the use of either isoosmolar or low-osmolar contrast media rather than high-osmolar contrast media particularly in patients at increased risk of CI-AKI. Older age, presence of atherosclerosis, congestive heart failure, chronic renal disease, nephrotoxic drugs, and diuretics may multiply the risk of CI-AKI.

## 1. Background

For many decades the high osmolality of contrast media has been regarded as the major factor responsible for acute renal failure following radiologic procedures [1–3].

Despite the availability of newer contrast media with lower osmolality, the incidence of contrast induced acute kidney injury (CI-AKI) still remains disappointingly high. Indeed, CI-AKI is the third cause of AKI that accounts for 10–13% of cases in hospitalized patients [4–6]. The increasing need of major interventional procedures and the older age of patients are important contributing factors for the high incidence of CI-AKI. Other factors that increase the risk of CI-AKI are comorbidities such as diabetes mellitus, chronic kidney disease (CKD), congestive heart failure, and atherosclerosis. There is a clinically relevant interplay between CI-AKI and presence of comorbidities; indeed, when there are no patient-related risk factors, the incidence of CIN is definitely lower (5%) [7]. In addition, the occurrence of CI-AKI in patients with comorbidities further increases the mortality risk, prolongs hospitalization stay, increases the costs, and worsens the prognosis.

The mechanisms responsible for CI-AKI are still poorly understood. Many of proposed pathogenic mechanisms of CI-AKI derive from experimental models that may not be directly translated into clinical setting [8].

In this review, the pathophysiologic mechanisms of CI-AKI will be analyzed and therapeutic options shortly commented on in the light of more recent available data.

## 2. Factors Responsible for the Hemodynamic and Tubular Changes Induced by Contrast Media

The changes occurring after administration of iodinated agents are not only the results of the direct action of contrast media on renal vasculature and tubular cells but also the results of hemodynamic and toxic changes due to presence of comorbidities such as CKD, diabetes, congestive heart failure, and/or use of diuretics, anti-inflammatory drugs, and nephrotoxic drugs before or in concomitance with contrast injection (Table 1). Indeed, volume depletion and low organ perfusion with concomitant hypoxia strongly affect renal hemodynamic and multiply the risk of CI-AKI [9–11].

### 2.1. Physicochemical Properties of Contrast Media

Contrast media can be categorized according to their osmolality in high-osmolar (approximately 1200 mOsm/kg), low-osmolar (600 to 800 mOsm/kg), and isoosmolar (290 mOsm/kg) contrast medium.

Newer contrast media with lower osmolality have been introduced and largely used in clinical practice during the last two decades in the effort to reduce the negative effects of osmolality on renal hemodynamics and incidence of CI-AKI. However, it is still debated whether there is a substantial difference between contrast media with different osmolality being in the literature present conflicting data. For instance, patients with CKD undergoing coronary angiography with the isoosmolar contrast medium iodixanol were found at lower risk of renal toxicity compared to those in whom the procedure was performed with the low-osmolar contrast medium ioxaglate [12, 13]. In addition, the incidence of CI-AKI was also significantly lower with iodixanol in those patients with more severe renal impairment, those with concomitant diabetes, those who received >140 mL of contrast, those with left ventricular ejection fraction >40%, and those aged <75 years [12]. Finally, in multivariate analysis, use of ioxaglate was an independent risk factor for CI-AKI [12]. A meta-analysis of pooled data from 2.727 patients indicated that use of isoosmolar contrast medium was associated with smaller rise in serum creatinine concentration and lower rate of CI-AKI compared to the rise observed after different low-osmolar contrast media [13]; better results were observed especially in high risk patients such as those with advanced CKD or CKD and concomitant diabetes [13]. In a post-hoc analysis of a randomized double-blind study comparing two contrast media (the low-osmolar iopamidol versus the isoosmolar contrast medium iodixanol) the incidence of adverse events was lower in patients who received the isoosmolar contrast medium [14].

A superiority of isoosmolar contrast media compared to low contrast media has not been confirmed by others. For instance, the rate of CI-AKI was not statistically different after administration of iopamidol or iodixanol in patients with moderate to severe CKD (eGFR 20 to 59 mL/min per 1.73 m^2^) with or without diabetes mellitus [15]. In a meta-analysis of 23 randomized controlled studies, no significant reduction was observed in the relative risk of CI-AKI with the use of iodixanol compared with nonionic low-osmolar contrast media pooled together [16]; in addition, no difference in reducing the risk of CI-AKI was found in high-risk patients between all studied contrast media [8–16]. In a very large multicenter randomized study enrolling more than 50.000 patients undergoing coronary procedures performed with either iodixanol or ioxaglate, the incidence of clinically significant renal failure was greatest for patients receiving the isoosmolar medium iodixanol [17]; the odds ratio of developing CI-AKI was significantly higher for either diabetic patients or patients with preexisting CKD [17].

Despite the available data which are largely discrepant and unable to demonstrate a clear superiority of one new contrast medium, all guidelines suggest to use either isoosmolar or low-osmolar rather than high-osmolar contrast media particularly in patients at increased risk of CI-AKI [10, 18, 19].

### 2.2. Volume of Contrast Media and Route of Injection

Volume of injected contrast media and route of administration are regarded as critical factors that may be responsible for the hemodynamic and tubular changes induced by contrast media.

Many decades ago it was observed that the incidence of CI-AKI was related to the volume of contrast injected and that the adherence to a formula to limit the use of contrast material significantly reduced the rates of CI-AKI [20]. Indeed, prospectively applying the maximal dose limit of 300 mL to 3322 patients undergoing coronary angiography, CI-AKI developed in only 2% of study population; CI-AKI developed in 21% of study population when dose was above the dose limit. Interestingly the reduction in the incidence of CI-AKI was attained with a dose not exceeding 300 mL, despite the fact that high-osmolar contrast media were used and the study population was represented by high risk patients [20].

Trials evaluating head-to-head the hemodynamic effects of intravenous and intra-arterial injection of contrast media are scarce. Data on this issue may be collected from some meta-analyses. The Contrast Media Safety Committee of the European Society of Urogenital Radiology systematically reviewed the results of papers published from 1996 to April 2010 that had evaluated the incidence of CI-AKI by individually comparing intravenous versus intra-arterial route. The Committee stated that the risk of CI-AKI was lower after intravenous than after intra-arterial route and that only patients with an estimated glomerular filtration rate less than 45 mL/min/1.73 m^2^ were at risk of CI-AKI after intravenous administration of contrast media [21]. Potential explanation could be the fact that in procedures requiring the intravenous route contrast medium is diluted into blood stream and slowly reaches the renal vasculature; in intra-arterial procedures contrast medium is minimally diluted and rapidly reaches kidneys.

Different results have been reported by another meta-analysis that analyzed data from studies performed to evaluate the effects of administration routes on the renal safety of isoosmolar iodixanol and pooled low-osmolar contrast media [22]. Lower risk of CI-AKI was observed after intra-arterial injection of iodixanol in patients who underwent interventional cardiology procedures; iodixanol was not associated with a reduction in CI-AKI with intravenous application.

The discrepancies observed even when the same contrast medium was administered suggest that interactions may exist between route of administration, physicochemical properties of contrast media, radiographic procedures, and study population. Patients who undergo procedures requiring intra-arterial injection likely have more clinically relevant comorbidities that *per se* amplify the risk of AKI. Finally, patient's hydration status may play as confounder.

Two studies underline the important role of study population and patients related risk factors. When intravenous (for computed tomographic angiography) versus intra-arterial (for digital subtraction angiography) route was compared within the same population the incidence of CI-AKI between the administration routes was not statistically significant [23]. In addition, no difference was found in patients who received both intra-arterial and intravenous contrast injections within one year after adjustment for patient-related risk factors such as age, gender, CKD, diabetes mellitus, congestive heart failure, hypertension, anaemia, and atrial fibrillation [24].

## 3. Hemodynamic Changes Induced by Contrast Media

**Figure 1:**
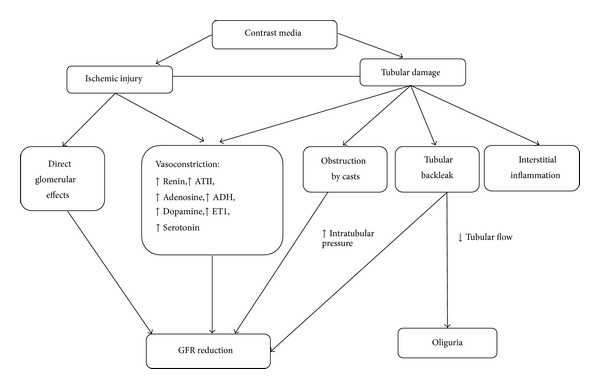
Cascade of events leading to contrast induced acute kidney injury.

The factors involved in the contrast media-induced renal vasoconstriction are reported in Figure 1.

The vasoconstriction induced by contrast media is the results of activation of vasoconstrictive mechanisms by one hand, and hampering or abolishing the action of vasodilating mechanisms by the other hand. Both mechanisms are amplified in presence of CKD and/or diabetes.

After contrast media injection, renal vasculature is more prone to vasoconstrictor stimuli, including angiotensin II, endothelin, and serotonin, and increased sensitivity to renal nerve stimulation [9]; in addition, vasodilator nitric oxide is reduced, while vasoconstrictive superoxides increase [9].

The hemodynamic changes induced by iodinated agents have been evaluated in experimental and clinical studies.

In dogs the vasoconstriction is the main factor responsible for prolonged reduction of glomerular filtration rate. Indeed, the injection of iodinated contrast media directly into renal artery of dogs causes an initial short-lasting vasodilatation, involving the vasculature of all organs; only in the kidneys the initial vasodilatation is followed by a prolonged vasoconstriction [25]. Injection of iothalamate in the left ventricle of dogs increased cardiac output and decreased the renal blood flow by 25%; in contrast, injection of isoosmolar volume of mannitol in the same experimental group increased cardiac output as well as renal blood flow. These findings suggest that the changes in renal hemodynamics are dependent on the characteristics of the contrast medium [26].

In humans the hemodynamic changes are similar to those observed in experimental studies. Renal blood flow shortly increases after radiocontrast injection; this increase is followed by a prolonged decrease that lasts up to 60 minutes. The decrease of blood flow is paralleled by a decrease of glomerular filtration rate. As for animal models, the biphasic hemodynamic response after contrast media injection is unique for the kidney.

The contrast media-induced vasoconstriction leads to marked reduction of oxygen delivery. The outer medulla is the portion of kidney mostly interested by reduced oxygen supply because it is directly vasoconstricted by contrast media and it is far from the descending vasa recta which deliver blood to it. Furthermore, hypoxia may be also caused by reduction of red blood cell velocity and increased red blood cell aggregation in renal medullary vessels as it has been shown in rats. Contrast media, namely, diatrizoate, iopromide, iohexol, and ioxaglate, iotrolan, were given in iodine equivalent doses (1600 mg/kg body wt). Mannitol (950 mOsm/liter) and Ringer's solution were used as controls. The same rat vessels were studied 30 minutes before and 30 minutes after injections. All contrast media and mannitol caused both red cell aggregation and cessation of blood flow [27]. Reduction of red blood cell velocity and increased red blood cell aggregation in renal medullary have been also observed in rats treated with either low or isoosmolar contrast media with low or high ionic ratio (ioxaglate: ionic ratio 3.0; iopromide: nonionic ratio 3.0; iotrolan: nonionic ratio 6.0) [28].

Medullary congestion and hypoxia activate the tubuloglomerular feedback mechanism and decrease GFR [29].

### 3.1. Mediators of Renal Vasoconstriction

Contrast media-induced vasoconstriction has been regarded as calcium-dependent phenomenon. Some data from experimental and clinical studies are in keeping with this hypothesis. Indeed, the vasoconstriction and the consequent reduction of glomerular filtration rate were significantly attenuated in magnitude and duration in dogs pretreated either with calcium channel blockers (verapamil, diltiazem), or with the calcium chelator EDTA [30]. In subjects with normal renal function undergoing urography with high-osmolar contrast agent diatrizoate the administration of nifedipine prevented the reduction of glomerular filtration rate and renal plasma flow [1, 31]. A 3-day treatment with the calcium channel blocker nitrendipine (20 mg/day, starting 1 day before X-ray examination) in patients with close to baseline normal renal function avoided the reduction of inulin clearance following intravascular administration of contrast media, while control patients showed a significant (27%) reduction in GFR on day 2 after contrast media injection [32]. Opposite results have been reported by other clinical studies. No significant difference in incidence of CI-AKI was observed in CKD patients on chronic nifedipine therapy after intravascular injection of contrast media compared to patients not on treatment with nifedipine [33]. Patients pretreated with a single 10 mg dose of nifedipine had similar change in serum creatinine concentration within 48 h of contrast media administration compared to no treatment group [34]. These conflicting results may be due to heterogeneity of patients enrolled, differences in radiological procedures, doses of contrast media administered, baseline renal function, and comorbidities. Likely calcium channel blockers may prevent contrast vasoconstriction and consequent reduction of renal blood flow and glomerular filtration rate in patients with close to normal renal function undergoing non invasive procedures [1, 31].

Calcium channel blockers are not among the pharmacological prevention strategies of CI-AKI suggested by recent guidelines [18].

Contrast dependent vasoconstriction may be induced by intrarenal renin-angiotensin system. This system is activated by both ischemia and increased sodium delivery to distal tubule due to osmotic property of contrast media. Intraglomerular pressure and consequently glomerular filtration rate are normally regulated by afferent and efferent arterioles tone. In presence of decreased renal blood flow, intraglomerular pressure is maintained by vasodilation of the afferent arteriole and vasoconstriction of the efferent arteriole. The latter is regulated by the intrarenal renin-angiotensin system.

The role of renin-angiotensin system in the vasoconstrictive response to contrast medium has been evaluated in experimental and clinical studies by using angiotensin converting enzyme inhibitors (ACE-i).

ACE-i reduce the constriction of efferent arteriole and increase the blood supply to medulla. Reduction of ischemia hampers ROS formation and consequently reduces the risk of CI-AKI [35].

High-osmolar contrast medium diatrizoate was injected into the renal artery of sodium-deplete and sodium-replete dogs [36]. Renal blood flow significantly decreased from baseline in sodium-deplete and sodium-replete dogs. The duration of the vasoconstriction phase was significantly prolonged in the sodium-deplete dogs. Blockade of the intrarenal renin-angiotensin system with infusion of Saralasin did not significantly alter the magnitude of vasoconstrictive response but decreased the duration of the vasoconstrictive phase in sodium-deplete animals.

Rate of CI-AKI dramatically decreased in patients treated with captopril (25 mg TID for 3 days, starting 1 h prior to contrast administration) compared to not treated patients [37]. In contrast, serum creatinine concentration did not change in patients with chronic renal insufficiency treated with ACE-i compared with not treated control group [38]. However, ACE-i may have also deleterious effects on renal hemodynamics [39]. ACE-i increased the risk of CI-AKI in patients with CKD undergoing coronary angiography. In multivariable analysis ACE-i treatment was powerful risk predictor of CI-AKI amongst well-known AKI predictors such as coronary artery disease, hypoalbuminemia, diabetes mellitus, reduced GFR, and congestive heart failure [39].

It should be taken in account that ACE-i and angiotensin receptor blockers decrease intraglomerular pressure by selective inhibition of angiotensin II-mediated vasoconstriction at the efferent arteriole. Therefore, these drugs will further decrease intraglomerular pressure in presence of reduced renal perfusion due to volume depletion [40–43].

Because of the opposite results reported by clinical study, recent guidelines suggest that ACE-i should not be administered for the prevention of CI-AKI and should be withdrawn before contrast media injection [21].

## 4. Renal Tubular Changes Induced by Iodinated Agents

Besides severe changes of systemic and renal haemodynamics, the infusion of contrast media has negative effects on renal tubular cells. Mainly the proximal tubules are affected by contrast media.

Tubular damage may be due to ischemia consequent to vasoconstriction and/or to direct cytotoxiticy of contrast media. There is an interplay between vasoconstriction and direct cytotoxiticy.

Intrarenal vasoconstriction decreases glomerular filtration pressure; tubular damage leads to formation of casts that obstruct tubuli and increases intratubular pressure; tubular back leakage decreases tubular fluid flow.

Renal tubular changes induced by contrast media have been evaluated in *in vivo* and *in vitro* experimental models.

In Wistar rats, the intravenous injection of two contrast media with similar physicochemical properties iobitridol and iohexol caused moderate to prominent alterations of lysosomes of the proximal convoluted tubular cells 2 hours after injection [42]. After 48 h, the changes induced by iobitridol had almost disappeared, whereas the iohexol group still showed a statistically significant vacuolization. No alterations were observed in control animals that received physiologic saline. These findings suggest that although the general physicochemical properties of iobitridol and iohexol appear similar *in vitro*, the different lysosomal alteration might reflect differences in their characteristics *in vivo*.

The cytotoxic effects of contrast media have been assessed by studying both tubular cytotoxiticy and alteration of signalling molecules in cultured human renal proximal tubular epithelial cell line. In this model osmolality of contrast media played a crucial role in cellular survival, growth, and proliferation. The cytotoxiticy on tubular cell was higher in cells exposed to high-osmolar contrast compared to those exposed to low-osmolar one. The alterations of signalling molecules was found greater in cell incubated with high-osmolar contrast medium [44, 45]. Using the same experimental model, either low-osmolar or isoosmolar contrast medium decreased phosphorylation process, affected iNOS expression and induced apoptotic processes; these alterations had deleterious effects on vasodilating mechanisms and on cellular survival, growth, and proliferation [46].

Tubular damage by contrast media has been assessed through urinary enzyme excretion in clinical studies. Enzymuria is able to detect the inception and the evolution of CI-AKI. Lipocalin is the most studied protein in the setting of CI-AKI being earlier marker of ischemic/toxic insults in CI-AKI than serum creatinine. Lipocalin is highly increased and excreted in the urine by direct activation of the transcription factor NF*κ*B that is involved in cell apoptosis [44, 45, 47–50].

### 4.1. Role of Reactive Oxygen Species in Tubular Cell Apoptosis

Reactive oxygen species (ROS) are involved in the pathophysiology of CI-KI. Contrast media enhance hypoxia and critically increase the production of ROS within the kidney [20]. ROS cause tubular and vascular endothelial cell injury. The increased oxidative stress in turn intensifies cellular hypoxia through endothelial dysfunction and alteration of mechanisms regulating tubular cells transport. These changes lead to marked tubular cell apoptosis. In an *in vitro* study [51] renal tubule epithelium cell line from normal rat (NRK-52E) was exposed to either increasing concentration of ioversol (a nonionic contrast media) or mannitol with the same osmolality as ioversol (420 mOsm kg^−1^); intracellular ROS production was assessed. Ioversol induced NRK-52E cells apoptosis in a concentration- and time-dependant manner via an increase in oxidative stress; in contrast mannitol had no effects. Irbesartan, a selective AT1 receptor antagonist with demonstrated antioxidative activity, attenuated the ioversol-induced apoptosis. These findings may suggest that prevention of CI-AKI could be based either on inhibition of ROS generation or increased ROS scavenging. Some clinical studies support this possibility, demonstrating a protective effect of N-acetyl cysteine by ROS scavenging or by reduced ROS generation [47–50].

## 5. Conclusions

Contrast media are responsible for hemodynamic and tubular changes that can cause CI-AKI. Despite the availability of newer contrast media, the incidence of CI-AKI remains disappointingly high. Major interventional procedures are frequently performed in older patients who have clinically relevant comorbidities. Avoiding all causes/factors of volume depletion and nephrotoxic drugs administration before any interventional radiologic procedure requiring injection of contrast media is the main tool to prevent or reduce the risk of CI-AKI.

## Figures and Tables

**Table 1:** Patient and procedure related potential risk factors responsible for contrast induced acute kidney injury.

Patient related	Procedure related
Chronic kidney disease	Major interventional procedures
Diabetes	Routes of administration
Congestive heart failure	Osmolality of contrast medium
Age > 70 years	Volume of contrast medium
Hypovolemia	Repeated doses of contrast medium
Nephrotoxic agents	
Anti-inflammatory drugs	
Atherosclerosis	
